# Health Service Seeking Behavior among Migrant Workers in Small and Medium-Sized Enterprises in Guangdong, China: Does Family Migration Matter?

**DOI:** 10.1155/2018/3620436

**Published:** 2018-11-21

**Authors:** Yuewen Dang, Guanyang Zou, Boli Peng, Li Ling

**Affiliations:** ^1^Faculty of Medical Statistics and Epidemiology, School of Public Health, Sun Yat-Sen University, Guangzhou 510080, China; ^2^Sun Yat-Sen Center for Migrant Health Policy, Sun Yat-Sen University, Guangzhou 510080, China; ^3^Institute for Global Health and Development, Queen Margaret University, Edinburgh EH21 6UU, UK

## Abstract

**Objective:**

This study aims to understand the health service seeking behavior of migrant workers and explore its association with their living status (i.e., living with family members or not), in Guangdong, China.

**Methods:**

This was a cross-sectional survey conducted with 912 migrant workers in 2012 using a structured questionnaire adapted from the National Health Service Survey. Data were analyzed using the multivariable logistic regression.

**Results:**

Of all migrant workers, 58% lived with at least one family member in the host city. Most of the respondents rated their health status being “very good or good” (58%). Fifty-four percent of the respondents reported having at least one disease in the past 12 months. Sixty-two percent of those who reported at least one disease visited doctors in the past 12 months. Of these, 22% returned to their hometown for medical treatment. Logistic regression showed that migrant workers living with families rated themselves as having better health status (*P*<0.05) but had more diseases (*P*>0.05) and had higher doctor visitation rate than those living with alone (58% vs. 66%,* P*<0.05).

**Conclusion:**

The Andersen health service utilization model helps to understand the health seeking behavior of the migrant workers in the host cities. Migrant workers living with family members were positively associated with self-rated health status and health service seeking behavior in small and medium-sized enterprises. Our findings suggest the importance of the assistance programs and social support to improve seeking of healthcare services among migrant groups, especially those who live alone in the host cities.

## 1. Introduction

Over the past three decades, China's reform and opening policy has dramatically promoted economic growth, contributing to the largest internal migration in history [1]. By 2016, the number of migrant workers had reached 263 million, an increase of 7.3% from 2012, accounting for about 20.5% of the total population of China [2–4]. Despite their great contribution to the development of the urban and national economy, migrants have remained a vulnerable group. Migrant workers often have unstable and insecure work associated with poor working conditions, occupational hazards, and long working hours [4]. Migrant workers are thus more vulnerable to occupational injuries, infectious diseases, and other health-related problems as compared to the local residents [5–7]. China's national policies have long been formulated on the household registration (*hukou*) system. Certain rights can only be granted to people who have local household registration (*hukou*), such as free education and social welfare. Given the great difficulty of transferring household registration, migrant workers rarely benefit from public medical insurance and assistance, with a higher out-of-pocket expense for medical services in host cities [8].

Many studies have reported poor health service seeking behavior among migrant workers in China. For instance, Mou and colleagues reported that only 38% of migrant workers sought health services upon being ill [9]. Insufficient seeking for healthcare may lead to inappropriate or delayed healthcare resulting in undesirable outcomes, such as high prevalence of infectious diseases and psychological disorders [10, 11]. Furthermore, the poor health service seeking behaviors of migrants would increase the burdens on healthcare resources and the health care delivery system in the destination cities. At this point, more attention should be paid to the health-seeking behavior of migrant workers.

Studies have identified a number of factors that influence the health service seeking behavior of the migrants. For instance, a large study in Shenzhen, one of the major destination cities of migrants, reported that insured migrants were more likely to seek health services than those uninsured (OR=1.44) [9]. Research conducted in Beijing, the capital of China, suggested that low income and long working hours were the most important factors of health service seeking behavior [12]. Migrants with the average monthly household income per capita of 251-500 RMB, 500-750RMB, 751-1000RMB, and 1001-1250RMB had 1.57 times, 1.78 times, 1.84 times, and 1.91 times the likelihood of seeking health service, respectively, as compared to those with the monthly household income per capita less than 250RMB. The probability of seeking health services was decreased by 41.3% if migrants worked more than 13 hours per day (OR=0.59), compared to those working less 8 hours per day [12]. In addition, factors like self-rated health status (OR=1.24) and reported chronic diseases (OR=3.50) were found to be the major predictor of seeking health service among migrants [9, 13]. Studies found that elderly migrants living in the host city for 10 or more years (OR=0.88) were less likely to seek health service [14]. The knowledge of healthcare services might also play an important role in the health service seeking behavior of migrant workers. Previous studies showed that migrants were more likely to use private services for general health care and delivery care than local residents (OR=1.86) due to low knowledge of public services, such as services of high-level hospitals and township hospitals [15]. Understanding these potential determinants of health services seeking behavior will be crucial to improving the health services utilization and health status of migrant workers in China.

In the recent years, there is an increasing trend that migrants move with the family members. In 2010, 70% of migrants lived with more than one family member in the host city and, of these, over 28% of them lived with all of their family members [16]. According to the Report on China's Migrant Population Development, a migrant family had an average of 2.61 people in their host city [17]. Previous studies have shown that migrants living with families, especially those living with the whole family, tended to have better socioeconomic status and remained more stable life than migrants living alone [16]. What is more, family members would help to facilitate the use of health services, from the aspects of providing information about healthcare system [18] and social support [19, 20]. Studies also found that migrants with a family presence in the destination (OR=4.88) were more likely to seek health service [21]. Therefore, we assume that migrants living with family members could be associated with better health service seeking behavior of the migrant workers.

On the basis of the Andersen health service utilization model [22], this paper aims to identify possible determinants influencing health service seeking behaviors among the migrant workers in small and medium-sized enterprises (SEMs) in Guangdong, China, and explore whether migrants living with families or not would be associated with the health service seeking behavior. We hope our study could offer useful information for future research and insight to tailor interventions to promote the health status of migrant workers.

## 2. Methods

### 2.1. The Conceptual Framework

**Figure 1:**
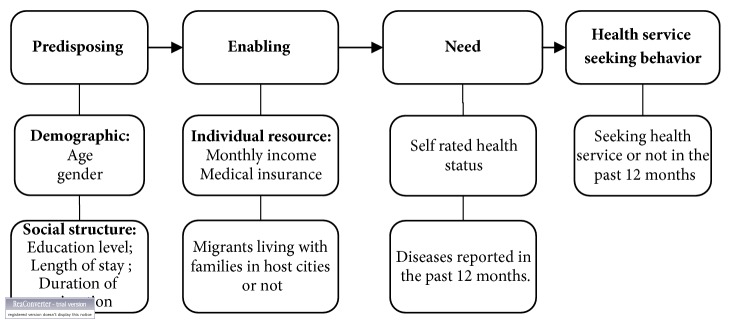
The modified Anderson health service utilization model including variables used in this study. Source: adapted from [23].

The Andersen behavioral model of health service utilization is a widely used framework to explain the health services utilization among populations [22]. The Andersen model was first constructed in 1968 and had gone through several phases of revision over the years [23]. The model applied in this research is Andersen's phase 1 model with some modifications. Therein the authors assumed that individuals' use of service is determined by three sequential clusters of factors: predisposing, enabling and need factors (Figure 1). Predisposing factors are defined as his or her predisposition to use health services, including demographic characteristics (e.g., age), social structures (e.g., education), and person's health beliefs (e.g., attitudes towards health services). The enabling factors include such variables as the availability of financial resources to pay for services, family support, and access to health insurance, which may enable or impede the use of health services. Perceived health status and reported diseases are included as need factors. The outcome variable is the seeking of health services.

### 2.2. Study Sites

A cross-sectional survey was conducted from August to October 2012 with a three-stage stratified sampling method in five cities of Guangdong province, a province located in southern China on the South China Sea coast. Guangdong, one of the most economically dynamic provinces in China, had the largest number of rural-to-urban migrants [4]. Based on economic and geographical representation and the proportion of migrants, two developed cities (Guangzhou and Foshan) and three less-developed cities (Zhaoqing, Qingyuan, and Shaoguan) were selected [24]. Then from the list of factories provided by the local government, we randomly selected 1-7 factories from each city using computer-generated random numbers. Finally, 912 respondents were selected from all the factories.

### 2.3. Sample Size Calculation

We estimated that 385 individuals were needed based on our previous study [1]

(1) $$\begin{array}{c} \left(\;n=\frac{Z_{\alpha /2}^{2}*\hat{P}*\left(1-\hat{P}\right)*N}{\delta^{2}*\left(N-1\right)+Z_{\alpha /2}^{2}*\hat{P}*\left(1-\hat{P}\right)}\;\right), \end{array}$$

where *δ* = 0.05 and *Z*_*α*/2_ = 1.96. According to the 2010 census, the total population of Guangdong province *N* was approximately 104 million [24]; $\hat{P}$ was the estimated rate of a doctor visiting in the past 12 months upon being ill (0.5). Then after adjusting the response rate (about 80%) and multivariable and multimodel analysis (about 1.7 times the univariate and single-model analysis), 819 (385×1.7/0.8≈819) individuals were eventually required to be recruited.

Participants who met the following criteria were recruited: (1) having nonlocal* Hukou;* (2) first-line workers working in manufactory plants; (3) leaving their* Hukou* registered cities for at least three months; (4) being able to provide informed written consent. We excluded workers who were management personnel or who had learning difficulties.

### 2.4. Survey Instruments

The questionnaire (see Appendix) was designed according to the contents of the China National Health Service Survey(NHSS), which has been organized by the Chinese Ministry of Health (MoH) every fifth year since 1993. During the process of questionnaire development, we invited experts in health service research for rigorous reviews and consultations. The questions in the questionnaire were divided into four parts according to Andersen's behavior model (Figure 1): predisposing factors, enabling factors, need factors, and outcome variable [25].

#### 2.4.1. Predisposing Factors

Predisposing factors included age (“≤20”, “20 – 29”, “30 – 39,” and “40-”), gender, education level, length of stay in the host city in years, and duration of migration (year). The length of stay was measured by the self-reported time interval (year) from the time when respondents first moved to the investigated city from their hometown till the time of survey. The duration of migration was measured by the self-reported calendar year and month in which the respondents first moved to another city from their hometown to search for a job.

#### 2.4.2. Enabling Factors

Enabling factors included monthly income (RMB), medical insurance coverage, and migrants living with at least one family member or not [21]. The medical insurance coverage was measured by an item asking respondents whether they had any types of medical insurance. Migrants living with family members or not were measured by a question whether there were family members who lived with you for more than six months in the host city [26, 27]. Monthly income (RMB) was divided into four levels (“<2000”, “2000-2499”, “2500-2999,” and “>=3000”) according to the distribution characteristics.

#### 2.4.3. Need Factors

The need factors included the variable of self-rated health status (SRH) and reported diseases. The self-rated health status was measured on a 5 point Likert scale from 5=very good to 1=very poor. Based on the SRH scale with five categories, the category of the “poor” and “very poor” was combined with the “fair” as poor SRH, and the category of “very good” was combined with the “good” as good SRH in regression analysis [28]. The reported diseases were measured by any diseases reported by the migrant workers about whether they had any diseases diagnosed by a physician or from their own opinion in the past 12 months according to a disease list given in the questionnaire. The disease list was a part of the Work Ability Index (WAI) scale which is an instrument used in clinical occupational health and research to assess work ability during health examinations and workplace surveys. WAI scale has been validated in the previous research also conducted by our team [1].

#### 2.4.4. Health Service Seeking Behavior

The outcome measurement of this study was the health service seeking behavior in the past 12 months. We estimate the percentage of participants who visited a doctor when having at least one reported disease (Yes/No). In addition, the variable of returning to the hometown for health services when falling ill was also included in this study.

### 2.5. Statistical Analysis

The database was constructed using Epidata 3.0 with double entry. All statistical analyses were performed using SAS statistical software (Version 9.4; SAS Institute Inc., Cary, NC, USA). To achieve our objectives, we reported the predisposing characteristics, enabling resources, need factors, and health service seeking behavior among all the respondents and by the groups of migrants living with families or not. All the individual characteristics were described as median (Q1, Q3) (Q1 stands for 25th percentile; Q3 stands for 75th percentile) and number (percentage) for categorical variables according to the groups of migrant workers with families or not. Chi-square and Wilcoxon rank sum test were conducted to assess the difference between these two groups of migrants. Multivariable logistic regressions were performed to explore the potential determinants of self-rated health status and health service seeking behavior. All potential confounding factors in the Andersen model (Figure 1) were included in the multivariable logistic regressions using enter methods. Considering the variation of the health seeking behavior in different investigation sites, the variables of the investigated city were also included in the final model. A 2-tailed alpha with* P*<0.05 was considered statistically significant. Unadjusted odds ratios (OR), adjusted odds ratios (AOR), and their 95% CIs were obtained to assess the association.

### 2.6. Ethics Approval

Ethical approval was obtained from Ethical Review Board of School of Public Health of Sun Yat-sen University. Written informed consent was obtained from all the study participants.

## 3. Results

### 3.1. General Characteristic of the Study Respondents

#### 3.1.1. Predisposing and Enabling Factors in the Andersen Behavioral Model

Of all 912 migrant workers, most (62%) were living in Guangzhou, and the proportion of migrants who lived with at least one family member in their host city was 58%. The average family size was 2.81±2.42 among the migrant workers who lived in host city.  Table 1 shows the predisposing and enabling factors, including demographics and social-economic characteristics by the groups of migrants with families or not. More than half of the migrant workers were younger than 30 years old (55%) and male (55%).

A large proportion of respondents was educated in secondary school (49%) and the median monthly income was 2200 (RMB) (Q1-Q3: 2000-3000). The median length of stay in the host city and the duration of migration were 3 years (Q1-Q3: 1.33-6.54) and 7 years (Q1-Q3: 3.00-13.00), respectively; eighty-eight percent of respondents had medical insurance.

Compared to the migrant workers living with families, migrants living alone were much younger, tended to be male, had a higher education level, and had a shorter stay in the host city and shorter duration of migration (*P*<0.05). The median monthly income and the percentage of medical insurance coverage were higher among the migrant workers living with families than those living alone, but there was no significant difference between these two groups (*P*>0.05).

#### 3.1.2. Needing Factors in the Andersen Behavioral Model

Nearly 60% of the respondents rated their health status as being “very good or good.” Fifty-four percent of all the migrant workers reported at least one disease in the past 12 months (Table 2). Compared to the migrant workers who lived without families in the host city, migrants who lived with families had better self-rated health status (*P*<0.05).

However, migrant workers living with families seemed to report slightly more diseases than those living alone in the past 12 months (mean ± SD: 1.62±2.38 vs. 1.55±2.35,* P*>0.05).

Multivariable logistic regression showed that migrant workers living with families were more likely to rate themselves as good health status, compared to those living alone (AOR=1.54, 95%CI: 1.12, 2.11). In addition, migrant workers who earned between 2000 and 2500 RMB per month were more likely to be good self-rated health status (AOR =1.64, 95%CI: 0.97, 2.76, and* P*=0.063), comparing to those with monthly income less than 2000 RMB. What is more, migrant workers who were reported at least one disease presented to be less likely to rate themselves as good health status than those reporting no diseases in the past 12 months (AOR = 0.43, 95%CI: 0.32, and 0.58) (Table 3).

#### 3.1.3. Outcome in the Andersen Behavioral Model: Health-Seeking Behavior of Migrant Workers in the Past 12 Months

Within the past 12 months, 303 (62%) migrant workers who reported at least one disease had sought healthcare. Of these, 68 (22%) selected to seek health services in their hometown. The self-reported main barriers for seeking health services among migrant workers upon sickness included the high cost of health services (52%), having no free time (30%), being a long distance away from medical institutions (9%), and lacking caregivers in the host city (7%) (Table 4). We did not find a significant difference between migrant workers living with families and those living alone in terms of seeking health service and the self-reported main barriers to seeking health service (*P*>0.05). However, among migrant workers who had sought health service in the past 12 months, migrants living alone tended to return to their hometown for health services (*P*<0.05).

### 3.2. Factors Influencing the Health Service Seeking Behavior

Univariate analysis showed that the variable of the length of stay in the host city, the duration of migration and self-rated health status were significantly associated with the health-seeking behavior of migrant workers in the past 12 months (*P*<0.05) (Table 5). Meanwhile, migrant workers living with families were more likely to seek health services (OR=1.40), compared to those living alone (*P*=0.07).

Further multivariable logistic models indicated that living with family members played an important role in health-seeking behavior. Compared to migrant workers living alone, respondents living with families were at 1.64-times higher chances of visiting a doctor in the past 12 months (AOR =1.64, 95%CI: 1.04, 2.60). Meanwhile, the chance of seeking health service would decrease by 48% as migrant workers rating themselves with good health status (AOR =0.52, 95%CI: 0.34, 0.79), compared to those reporting poor health status (Table 5).

## 4. Discussion

On the basis of the Andersen health service utilization model, this paper facilitates our understanding of the health service seeking behavior among migrant workers in SEMs. Most of the respondents rated their health status being “very good or good” (58%). Half (54%) reported having at least one disease in the past 12 months and more than half (62%) of these who reported at least one disease visited doctors in the past 12 months. Consistent with the trend of family migration [17, 29], this study showed that nearly 60% of the migrant workers lived with family members in their host city. Migrants living with families rated themselves as having better health status (*P*<0.05) but reported more diseases (*P*>0.05) and had higher doctor visitation rate than those living without families (58% vs. 66%,* P*<0.05).

Although more than half of the migrants reported a disease in the past 12 months, most (58%) of respondents rated themselves as being “very good or good” health status. This proportion was similar to that of migrant workers in a nationally representative survey in 2012 (66%) [30] and general migrants in Guangzhou (70%) and Beijing (68%) [31]. Our study also suggested that migrant workers living with families tend to have better perceived health than those living alone. This may be due to the better social support received [32] and stable family relationships in those migrants living with families [33]. However, despite the majority of migrants living with the families rating themselves as having good health status, still over half of these migrants reported at least one diagnosed disease in the past 12 months. Compared to the migrants who lived alone, migrants who lived with family reported slightly more diseases, although no significant difference was found between these two groups. Therefore, their objective health status was not necessarily better than that of the migrant workers living alone. Migrants may tend to overestimate their healthy status as they may have poor understanding of health due to the low level of education [34]. In our study, nearly 60% of the migrant workers were educated at or below the level of secondary school; most of the migrant workers who lived with the families were educated at or below the level of secondary school (64% versus 46% among migrants living alone).

We found that about 62% of migrant workers had visited a doctor when they fell sick in the past 12 months. Comparing with other literature is difficult, as most of other studies have used doctor visiting rate in the past two weeks. The previous studies found that 36% to 38% of migrant workers visited a doctor when they fell ill in the past two weeks [9, 12, 35]. Consistent with other studies [12, 13], the main barriers to seeking health services upon illness for migrant workers included high medical expenditure, time availability, distance to health facilities, and lack of caregivers in the host city. Due to the household registration system, migrants were rarely entitled to public medical insurance and assistance program in the host city, causing high out-of-pocket expenses for health service [36]. Therefore, migrants may prefer to return to their hometown to seek medical service upon illness since they may have health insurance in their hometown [13]. In our study, this proportion was up to 22% of the total respondents who had sought health service in the past 12 months.

Our results showed that migrant workers living with families were more likely to seek health services in the past 12 months than those living alone, after adjusting all potential confounding factors in the Andersen model. In addition, among migrant workers who had sought health service in the past 12 months, those living alone tended to return to their hometown for health services. There are a number of plausible explanations. Firstly, migrants living with families tended to stay in the host city for longer time [16]. This may increase their social networking than those who live alone in the host city and helps to improve their awareness and resources to seek health services in the host city [20]. Secondly, better attention to health could be partly explained by the intimacy between family members and a sense of responsibility among the family among those living with the families [37, 38]. Thirdly, family members of migrants would be the main sources of information regarding the local healthcare system, and this facilitates the health service seeking behavior of migrants [18]. Therefore, our study suggests the importance of the assistance programs and social support to improve seeking of health care services among migrant workers, especially those living alone. Understandably, we also found that health-seeking behavior was negatively associated with self-rated health status. Mirant workers who rated themselves as better health status were more likely not to seek health service [13, 39].

Our study has several limitations. Firstly, we did not assess the validity and applicability of the questionnaire; however, it has been put through rigorous reviews and repeated consultations with experts in health service research. Secondly, recall bias should exist in the questionnaire survey. On the one hand, respondents may underreport their illness and health-seeking behavior in 12 months. On the other hand, it is unclear whether the family members joined the migrant workers before or after the illness onset of the migrant workers. If the family members joined the migrant workers after the illness onset, the influence of families on the health service seeking behavior in the host city would be overestimated. However, the family members in this study were limited to those who lived with the respondents for at least six months in the host city. This helps to reduce the research bias to some extent. Thirdly, the interrelationships between different variables were not included in this study. Although the Anderson model has the potential for understanding the interrelationships between different variables, this paper only focuses on explaining the health seeking behavior of the migrant workers. This should be considered in the future studies to better understand the dynamics of the health service seeking behavior. Finally, although our findings might indicate the association between migrant workers living with family members or not in the host cities and their health-seeking behavior, the cross-sectional study design itself does not establish their causal relationship. A larger-scale, longitudinal investigation will help to establish the causal effect of migrant workers living with family members on seeking health care services in the future.

## 5. Conclusion

The Andersen health service utilization model helps to understand the health seeking behavior of the migrant workers in the host cities. Despite more than half of the migrant workers feeling positive about their health status, still more than half reported having at least one disease in the past 12 months; and among those who reported at least one disease, nearly 40% did not seek care from the doctor. Migrant workers living with family members were positively associated with self-rated health status and health service seeking behavior in small and medium-sized enterprises. Our findings suggest the importance of the assistance programs and social support to improve seeking of health care services among migrant groups, especially those living alone in the host cities.

## Figures and Tables

**Table 1:** Migrant workers' predisposing and enabling characteristics.

Variables	Total *N* (%)	Migrant workers living		**χ** ^2^ ***(Z)***	*P*-value
		Alone	With families *N* (%)		
		*N* (%)			
**Predisposing Factors**					
**Age (years), **median (Q1,Q3)	29 (22, 38)	24 (21, 31)	33 (26, 41)	-10.87	<0.01
**Age (years)**					
≤20	132 (14.68)	87 (23.26)	45 (8.57)	101.93	<0.001
20~30	367 (40.82)	192 (51.34)	175 (33.33)		
30~40	225 (25.03)	54 (14.44)	171 (32.57)		
40~	175 (19.47)	41 (10.96)	134 (25.52)		
**Gender**					
male	498 (54.85)	250 (66.49)	248 (46.62)	35.13	<0.01
female	410 (45.15)	126 (33.51)	284 (53.38)		
**Education level**					
primary school or illiteracy	69 (7.60)	18 (4.75)	51 (9.64)	45.96	<0.01
Secondary school	441 (48.57)	156 (41.16)	285 (53.88)		
High School or equivalent	318 (35.02)	148 (39.05)	170 (32.14)		
University/College or above	80 (8.81)	57 (15.04)	23 (4.35)		
**Length of stay in the host city (years), **median (Q1,Q3)	3.00	2.08	4.33	-9.28	<0.01
	(1.33, 6.54)	(0.83, 3.67)	(2.00, 8.83)		
**Duration of migration, **median (Q1,Q3)	7.00	5.00	9.00	-8.88	<0.01
	(3.00, 13.00)	(2.00, 8.00)	(4.00, 14.00)		
**Enabling Factors**					
**Monthly income (RMB), **median (Q1,Q3)	2200	2100	2300	-0.79	0.43
	(2000, 3000)	(2000, 3000)	(2000, 3000)		
**Monthly income (RMB)**					
<2000	181 (20.25)	83 (22.74)	98 (18.53)	4.35	0.23
2000-2499	330 (36.91)	129 (35.07)	202 (38.19)		
2500-2999	145 (16.22)	52 (14.25)	93 (17.58)		
>=3000	238 (26.62)	102 (27.95)	136 (25.71)		
**Medical insurance **					
No	105 (11.60)	52 (13.83)	53 (10.02)	3.11	0.08
Yes	800 (88.40)	324 (86.17)	476 (89.98)		

**Table 2:** Migrant workers' self-rated health status and reported diseases in the past 12 months.

Variables	Total *N* (%)	Migrant workers living		**χ** ^2^ ***(Z)***	*P*-value
		Alone	With families *N* (%)		
		*N* (%)			
**Self-rated health status **					
Very good	181 (19.96)	70 (18.57)	111 (20.94)	8.19	0.042
Good	344 (37.93)	128 (33.95)	216 (40.75)		
Fair	230 (25.36)	111 (29.44)	119 (22.45)		
Poor	152 (16.76)	68 (18.04)	84 (15.85)		
Very poor	0	0	0		
**Diseases Number** median (Q1,Q3)	1^a^ (0, 2)	1^b^ (0, 2)	1^c^ (0, 2)	-0.47	0.64
**Reported diseases**					
No	423 (46.38)	179 (47.23)	243 (45.78)	0.19	0.67
Yes	489 (53.62)	200 (52.77)	289 (54.22)		

*Note.* ^a^mean ± SD: 1.59±2.37; ^b^mean ± SD: 1.55±2.35; ^c^mean ± SD: 1.62±2.38.

**Table 3:** Logistic regression model on factors related to good self-rated health status among migrant workers.

Variables	*β*(SE)	Wald	AOR (95%CI)	*P*-value
**Living with families**				
Yes (No)	0.43 (0.16)	6.96	1.54 (1.12, 2.11)	<0.01
**Monthly income (RMB)**				
2000-2499 (<2000)	-0.19 (0.21)	0.78	0.83 (0.55, 1.26)	0.38
2500-2999 (<2000)	0.49 (0.27)	3.45	1.64 (0.97, 2.76)	0.063
>=3000 (<2000)	0.17 (0.25)	0.46	1.18 (0.73, 1.91)	0.50
**Reported diseases**				
Yes (No)	-0.845 (0.15)	31.95	0.43 (0.32, 0.58)	<0.01

*Note.* The variable in the parenthesis was the reference. Other variables included in the model were age, gender, education level, length of stay in host city, duration of migration, and medical insurance. All of these variables did not show statistical significance in the model (*P*>0.05).

**Table 4:** Health-seeking behavior and self-reported main barriers among migrant workers who reported at least one disease in the past 12 months.

Variables	Total *N* (%)	Migrant workers living		**χ** ^2^	*P*-value
		Alone	With families *N* (%)		
		*N* (%)			
**Health seeking **(n=487)					
No	184 (37.78)	85 (42.50)	99 (34.49)	3.21	0.07
Yes	303 (62.22)	115 (57.50)	188 (65.51)		
**Health service seeking in hometown **(n=303)					
No	235 (77.56)	82 (71.30)	153 (81.38)	4.16	0.041
Yes	68 (22.44)	33 (28.70)	35 (18.62)		
**Main barriers **(n=97)					
High cost of health services	50 (51.55)	16 (47.06)	34 (53.97)	—	0.93^d^
Having no free time	29 (29.90)	12 (35.29)	17 (26.98)		
Long distance from medical institutions	9 (9.28)	3 (8.82)	6 (9.52)		
Lacking caregivers in the host city	7 (7.22)	2 (5.88)	5 (7.94)		
Don't know where to go	2 (2.06)	1 (2.94)	1 (1.59)		

*Note.* ^d^Fisher's exact test was used to compare the distributions of variables between migrant living with families and those living alone.

**Table 5:** Logistic regression model on factors related to health service seeking behavior among migrant workers who fell sick in the past 12 months.

Variables	Number of sick	Number of those who saw a doctor (%)	Univariate Analysis	Final Model^e^
			OR (95% CI)	AOR (95% CI)
**Predisposing factors**				
**Age**				
≤20	82	51 (62.20)	1.00	1.00
20~30	194	116 (59.79)	0.90 (0.53, 1.54)	0.73 (0.39, 1.38)
30~40	117	81 (69.23)	1.37 (0.76, 2.48)	0.72 (0.31, 1.68)
40~	86	51 (59.30)	0.89 (0.48, 1.65)	0.44 (0.18, 1.11)
**Gender **				
Male	276	171 (61.96)	1.00	1.00
Female	209	131 (62.68)	1.03 (0.71, 1.49)	1.07 (0.66, 1.75)
**Education level**				
Primary school or illiteracy	30	19 (63.33)	1.00	1.00
Secondary school	219	139 (63.47)	1.01 (0.46, 2.22)	0.740 (0.28, 1.96)
High School or equivalent	195	119 (61.03)	0.91 (0.41, 2.01)	0.78 (0.28, 2.20)
University/College or above	41	24 (58.54)	0.82 (0.31, 2.15)	0.92 (0.26, 3.33)
**Length of stay (year) **	466	290 (62.23)	1.05 (1.01, 1.09)*∗*	1.02 (0.970, 1.08)
**Duration of migration (year)**	468	289 (61.75)	1.03 (1.00, 1.06)*∗*	1.03 (0.98, 1.08)
**Enabling Factors**				
**Monthly income (RMB)**				
<2000	94	55 (58.51)	1.00	1.00
2000-2499	177	119 (66.67)	1.42 (0.85, 2.38)	1.56 (0.85, 2.84)
2500-2999	70	44 (62.86)	1.20 (0.64, 2.27)	1.09 (0.51, 2.33)
>=3000	132	77 (58.33)	0.99 (0.58, 1.70)	1.02 (0.51, 2.01)
**Medical insurance**				
No	61	36 (59.02)	1.00	1.00
Yes	421	266 (62.95)	1.18 (0.68, 2.04)	1.40 (0.75, 2.61)
**Living with families**				
No	200	115 (57.50)	1.00	1.00
Yes	287	188 (65.51)	1.40 (0.97, 2.04)	1.64 (1.04, 2.60)*∗*
**Need Factor**				
**Self-rated health status**				
Poor	253	173 (68.38)	1.00	1.00
Good	234	130 (55.56)	0.58 (0.40, 0.84)*∗∗*	0.52 (0.34, 0.79)*∗∗*

*Note.∗P*<0.05, *∗∗P*<0.01, and *∗∗∗P*<0.001.

^e^Different investigation locations, the variable of the investigated city, were included in the final regression model using enter method (*P*=0.42>0.05).

## Data Availability

The dataset used to support this study are available from the corresponding author on reasonable request.

## Supplementary Materials

Supplementary Materials Main items of the questionnaire are included as an appendix in this paper. The questionnaire is part of the project titled Occupational Health Status of Migrant Workers in Small and Medium-sized Enterprises in Guangdong Province, China, a survey conducted by Sun Yat-sen Centre for Migrant Health Policy in 2012.
