# Supplementary material for: Health Service Seeking Behavior among Migrant Workers in Small and Medium-Sized Enterprises in Guangdong, China: Does Family Migration Matter?
Source: Biomed Res Int. 2018 Nov 21;2018:3620436. doi: 10.1155/2018/3620436 (PMC6288583; doi:10.1155/2018/3620436)
Supplement: Supplementary Materials — Main items of the questionnaire are included as an appendix in this paper. The questionnaire is part of the project titled Occupational Health Status of Migrant Workers in Small and Medium-sized Enterprises in Guangdong Province, China, a survey conducted by Sun Yat-sen Centre for Migrant Health Policy in 2012. [file 3620436.f1.pdf]

Appendix:

Survey of Occupational Health Status of Migrant Workers in Small and Medium-sized Enterprises in Guangdong Province, China

Part I Demographic characteristics

1. Birthday : year of birth :_______ month of birth :______ ;
2. Sex: □1 male □2 female
3. Where is the place of your household registration ('*hukou*')?

   _________county (district) _________city_________province
4. What is the highest level of education you have completed?

   □1 primary school or illiteracy □2 Secondary school

   □3 High School or equivalent □4 University/College or above
5. Were there family members who lived with you for more than six months in the host city?

   □1 No, I'm living alone

   □2 Yes and how many: ______.
6. Your monthly income from the job :_____________RMB/ per month

Part Ⅱ Migration

1. Length of stay in the investigated city :___year and ___month
2. When you firstly migrated from your home to cities : _______month ______year

Part Ⅲ Insurance

What kind of medical insurance do you have?

□1 New Rural Cooperative Medical Insurance

□2 Urban Employee Basic Medical Insurance

□3 Urban Resident Basic Medical Insurance

□4 Special insurance for the migrants

□5 Employment injury insurance

□6 Employment injury insurance

□7 Commercial health insurance

□8 Have one, but don’t know which one is

□9 None of those above

Part Ⅳ Health and health service utilization

1. In general, would you say your health is :

☐1 very good ☐2 good ☐3 fair ☐4 poor ☐5 very poor

2. Number of the current diseases diagnosed by a physician:

In the following list, mark your diseases or injuries for the past 12 months. Also indicated whether a physician has diagnosed or treated these disease. For each, disease, therefore, there can be 2, 1 or no alternative circled.

	Injury from accidents	Yes, physician’s diagnosis	Yes, own opinion
01	back	1	2
02	arm/hand	1	2
03	leg/foot	1	2
04	other parts of the body	1	2
	where and what :		
	**Musculoskeletal disease**	**Yes, physician’s diagnosis**	**Yes, own opinion**
05	disorder of the upper back or cervical, spine, repeated instances of pain	1	2
06	disorder of the lower back, repeated instances of pain	1	2
07	(sciatica) pain radiating from the back into the leg	1	2
08	A mulsculoskeletal disorder affecting the limbs (hands, feet), repeated instances of pain	1	2

09	rheumatoid arthritis	1	2
10	other musculoskeletal disorder	1	2
	what :		
	Cardiovascular diseases	Yes, physician's diagnosis	Yes, own opinion
11	hypertension (high blood pressure)	1	2
12	coronary heart disease, chest pains during exercises (angina pectoris)	1	2
13	coronary thrombosis, myocardial infarction	1	2
14	cardiac insufficiency	1	2
15	other cardiovascular diseases	1	2
	what :		
	Respiratory disease	Yes, physician's diagnosis	Yes, own opinion
16	repeated infections of the respiratory tract (also tonsillitis, acute sinusitis, acute bronchitis)	1	2
17	chronic bronchitis	1	2
18	chronic sinusitis	1	2
19	bronchial asthma	1	2
20	emphysema	1	2
21	pulmonary tuberculosis	1	2
22	other respiratory diseases	1	2
	what? :		
	Mental disorder	Yes, physician's diagnosis	Yes, own opinion
23	mental disease or severe mental health problem (for example,	1	2

	severe depression, mental disturbance)		
24	slight mental disorder or problem (for example, slight depression, tension, anxiety, insomnia)	1	2
	Neurological and sensory disease	Yes, physician’s diagnosis	Yes, own opinion
29	problems or injury to hearing (for example epicophosis, tinnitus, acute or chronic otitis media, myringorupture)	1	2
30	visual disease or injury (for example glaucoma, cataract, trachoma)	1	2
31	neurological disease (for example stroke, neuralgia, migraine, epilepsy)	1	2
32	other neurological or sensory disease	1	2
	what :		
	Digestive disease	Yes, physician’s diagnosis	Yes, own opinion
35	gallstones or disease	1	2
36	liver or pancreatic disease	1	2
37	gastric or duodenal ulcer	1	2
38	gastritis or duodenal irritation	1	2
39	colonic irritation, colitis	1	2
40	other digestive diseases	1	2
	what :		
	Genitourinary disease	Yes, physician’s diagnosis	Yes, own opinion
41	urinary tract infection	1	2
42	kidney disease	1	2

43	genitals disease (for example fallopian tube infection in women or prostatic infection in men	1	2
44	other genitourinary diseases	1	2
	what :		
	Skin disease	Yes, physician’s diagnosis	Yes, own opinion
45	allergic rash, eczema	1	2
46	other rashes	1	2
47	what :	1	2
48	other skin diseases	1	2
	what :		
	Tumor	Yes, physician’s diagnosis	Yes, own opinion
49	benign tumor	1	2
50	malignant tumor (cancer)	1	2
	what and where :		
	Endocrine and metabolic diseases	Yes, physician’s diagnosis	Yes, own opinion
51	obesity	1	2
52	diabetes	1	2
53	goiter or others thyroid disease	1	2
54	other endocrine or metabolic disease	1	2
	what :		
	Blood diseases and birth defects	Yes, physician’s	Yes, own

		diagnosis	opinion
55	anemia	1	2
56	other blood disorder	1	2
	what :		
57	birth defect	1	2
	what :		
58	Other disorder or disease	1	2
	what :		

3. In the past 12 months, have you seen a doctor from your practice (not included the purchase of medicine in the pharmacy)?

   □1 No   □2 Yes ➡ 3.1 Have you seen a doctor back to your hometown?

   □1 No   □2 Yes

4. What's the barriers you didn't go to see the doctor when you were ill:

   Main barrier ______ (only one option);   Secondary barrier ______ (only one option)

   □1 High cost of health services   □2 Lacking caregivers in the host city

   □3 Having no free time   □4 Long distance from medical institutions

   □5 Don't know where to go
